# A {Co_9_}-Added Polyoxometalate for Efficient Visible-Light-Driven Hydrogen Evolution

**DOI:** 10.3390/molecules28020664

**Published:** 2023-01-09

**Authors:** Zhen-Wen Wang, Guo-Yu Yang

**Affiliations:** MOE Key Laboratory of Cluster Science, School of Chemistry and Chemical Engineering, Beijing Institute of Technology, Beijing 102488, China

**Keywords:** lacunary directing strategy, polyoxometalates, hydrothermal syntheses, visible-light-driven hydrogen evolution

## Abstract

A polyanion cluster H_6_Na_8_Cs_3_[Co_9_(μ_3_-OH)_3_(H_2_O)_6_(HPO_4_)_2_(B-α-PW_9_O_34_)_3_]Cl·40H_2_O (**1**) was made with the guidance of the lacunary directing strategy under hydrothermal conditions. Compound **1** was characterized by single-crystal X-ray diffraction, powder X-ray diffraction, and thermogravimetric analysis, respectively. Single-crystal X-ray diffraction analyses showed that **1** consists of three anions [B-α-PW_9_O_34_]^9−^ and a cyclic cationic [Co_9_(μ_3_-OH)_3_(H_2_O)_6_]^15+^ and two anions HPO_4_^2−^. Variable-magnetic properties indicate antiferromagnetic interactions in **1**. Visible-light-driven hydrogen evolution tests demonstrated that **1** was an efficient water reduction catalyst with an H_2_ evolution rate of 1217.6 μmol h^−1^ g^−1^.

## 1. Introduction

Polyoxometalates (POMs) have attracted increasing attention due to their intriguing structural diversity [1] and diverse applications in drugs [2,3], magnetism [4,5], and catalysis [4,5,6,7,8,9,10,11]. Lacunary POMs as precursors can induce transition metal (TM) cations’ aggregation to form TM oxo-clusters under hydrothermal conditions, producing a class of TM-added POMs (TMAPs) with interesting physicochemical properties [12,13,14,15]. Up to now, hydrothermal synthesis was successfully used in creating a large number of TMAPs. Since 2007, we have reported a large number of organic–inorganic hybrid Ni_6_-added POMs under the guidance of the lacunary directing strategy, using the lacunary sites of tri-lacunary POMs fragments {XW_9_O_34_}^n−^ (X = P/Si/Ge, *n* = 9, 10) as structure-directing agents (SDAs) to induce the TM-oxo clusters aggregation to form novel TMAPs [16,17,18,19,20,21].

POMs have unique redox properties and modifiable Lewis acidity but their inherent poor stability and tendency to aggregate in solution have hindered further development [20]. TMAPs are widely used in photocatalysis [22,23] and electrocatalysis [24] fields due to their physicochemical properties and reversible multi-electron transfer ability. TMAPs have been widely studied due to their unique structural diversity, magnetic properties, and catalytic properties. Taking the above into account, TMAPs have demonstrated a massive potential for application for visible-light-driven Hydrogen evolution as heterogeneous water reduction catalysts (WRCs) [14,15,25].

Co-containing complexes were applied in photocatalytic water-splitting reactions with good reactivity [26] and Co^2+^ can be induced by tri-lacunary POMs fragments {XW_9_O_34_}^n−^, thus improving the performance of Co-added POMs (CoAPs) as the catalytic active center in visible-light-driven H_2_ evolution. Herein, a polyanion (HPO_4_)_2_@-{Co_9_(PW_9_)_3_} cluster was made with the guidance of the lacunary directing strategy under hydrothermal conditions. Interestingly, the HPO_4_ groups of **1** originate from the central heteroatom of the tri-lacunary precursor [A-*α*-PW_9_O_34_]^9−^ and assemble with the cyclic (HPO_4_)_2_@{Co_9_} cluster to form CoAP. Moreover, visible-light-driven H_2_ evolution tests have demonstrated that CoAP is an efficient WRC with the H_2_ evlution rate of 1217.6 μmol h^−1^ g^−1^, indicating that **1** is a potential heterogeneous WRC.

## 2. Experimental Section

### 2.1. General Procedure

All chemicals were acquired commercially and used without further purification. The synthetic method of the Na_10_[A-α-PW_9_O_34_]·7H_2_O ({PW_9_}) was derived from previous literature methods [27]. Powder X-ray diffraction (PXRD) patterns were recorded on a Bruker D8 Advance XRD diffractometer (Karlsruhe, Germany) with Cu Kα radiation (*λ* = 1.54056 Å). FT-IR spectra were measured by using a Thermo Scientific Nicolet iS10 FT-IR spectrometer (Waltham, MA, USA) in the range of 400–4000 cm^−1^ with KBr pallets. Thermogravimetric analyses were conducted under air flowing on a Mettler−Toledo TGA/DSC 1000 (Zurich, Switzerland) with a heating rate of 10 °C min^−1^ from 25 to 1000 °C. UV–Vis absorption spectra were obtained using a Shimadzu UV3600 spectrometer (Kyoto, Japan).

### 2.2. Synthesis of ***1***

Co(NO_3_)·6H_2_O (0.536 g, 1.84 mmol), {PW_9_} (0.597 g, 0.233 mmol), and CsCl (0.308 g, 1.829 mmol) were mixed in 8 mL H_2_O and 5 drops acetic acid. After stirring for 1 h, the mixed solution was transferred to a 25 mL Teflon-lined autoclave and kept at 80 °C for 24 h. When the reaction was complete and cooled to room temperature, the product was filtered and the hexagonal pink crystals were washed with distilled water in a yield of 0.052 g (0.058 mmol), a yield of 7.51% (based on {PW_9_}). IR data (KBr pellet, cm^−1^): 3430 (s), 1630 (m), 1030 (s), 933 (s), 883 (s), 806 (s), 721 (s).

### 2.3. X-ray Crystallography

A suitable crystal was selected and put on a Bruker APEX-II CCD diffractometer. This crystal was kept at 296.15 K during data collection. Using Olex2 [28], the structure was solved with the ShelXT [29] structure solution program using Intrinsic Phasing and refined with the ShelXL [30] refinement package using Least Squares minimization. The contribution of these disordered solvent molecules to the overall intensity data of the structure was treated using the SQUEEZE method [31] in PLATON (25 lattice water molecules in **1** are estimated by TGA). The crystallographic data structure refinement is listed in Table 1. Detailed crystallographic data of the title crystal were deposited on the Cambridge Crystallographic Data Centre with CCDC reference number 2213769 for **1**. These data can be obtained free of charge via www.ccdc.cam.ac.uk/conts/retrieving.html accessed on 29 June 2022, or by emailing data_request@ccdc.cam.ac.uk, or by contacting The Cambridge Crystallographic Data Centre, 12 Union Road, Cambridge CB21EZ, UK; Fax: +44-1223 336 033.

## 3. Result and Discussion

### 3.1. Structure of ***1***

**Figure 1:**
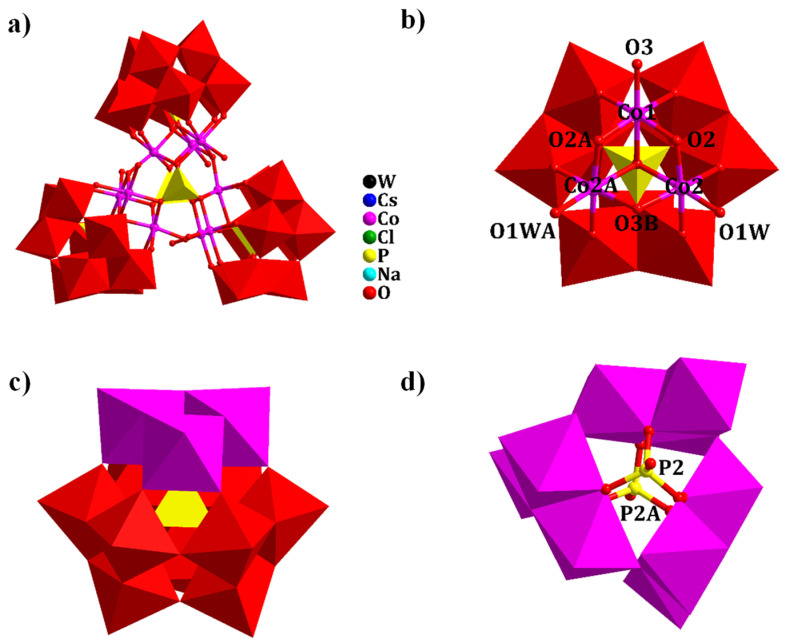
(**a**) View of polyoxoanion of **1**; (**b**) the coordination environment of [Co_3_O_2_(μ_3_-OH)_2_(H_2_O)_2_- (B-α-PW_9_O_34_)]^9−^. Symmetry codes: A (x, y, 1.5 − z), B (1 − y, 1 + x − y, z); (**c**) polyhedral view of [Co_3_O_2_(μ_3_-OH)_2_ -(H_2_O)_2_(B-α-PW_9_O_34_)]^9−^; (**d**) the {Co_9_} cluster capped by two HPO_4_ groups.

The structure of **1** crystallized in the hexagonal space group *P*6_3_/*m*. The structure contains one [Co_9_(μ_3_-OH)_3_(H_2_O)_6_(HPO_4_)_2_(B-α-PW_9_O_34_)_3_]^16−^ (abb. as (HPO_4_)_2_@{Co_9_(PW_9_)_3_}, Figure 1a) cluster, three Cs^+^, eight Na^+^ and 15 water molecules. Bond valance sum (BVS) calculations show that the valence of W, Co, O, Cl and P atoms are +6, +2, −2, −1 and +5, respectively. The calculations reveal low values for O1 (1.299) and O3 (1.084) demonstrating that O1 and O3 further bond to H^+^ as OH groups (Table S1, Supplementary Materials). The polyanion (HPO_4_)_2_@{Co_9_(PW_9_)_3_} cluster can be viewed as consisting of three [Co_3_O_2_(μ_3_-OH)_2_(H_2_O)_2_(B-α-PW_9_O_34_)]^9−^ (abb. as {Co_3_PW_9_}) units (Figure 1b) and two HPO_4_ tetrahedra. Co^2+^ are six-coordinate octahedral configurations with Co–O bond lengths ranging from 1.998 to 2.182 Å. In addition, two Co^2+^ (Co2, Co2A) have coordinated water molecules (O1W, O1WA), and the other Co^2+^ (Co3) carries a *μ*_3_-OH (O3, O3B) in the Co_3_O_13_ ({Co_3_}) cluster (Figure 1b). Three Co^2+^ share edges to form a triangular {Co_3_} cluster, and the {Co_3_} cluster cap on the [B-α-PW_9_O_34_]^9−^ fragment to form the {Co_3_PW_9_} unit (Figure 1b,c). Three {Co_3_PW_9_} units are connected to each other through three *μ*_3_-OH forming a cyclic {Co_9_(PW_9_)_3_} unit. Two openings existed in the cyclic {Co_9_(PW_9_)_3_} unit, in which each consists of three O atoms forming a triangle with a side length of 2.501 Å and matching the size of the HPO_4_^2−^ tetrahedron (Figure 1d). The two HPO_4_ units are capped on both sides of the ring to form a cluster (HPO_4_)_2_@{Co_9_(PW_9_)_3_} (Figure 1a). Interestingly, [Cl@{Cs_3_(H_2_O)_6_}] (Figure S1, Supplementary Materials) fills the middle of the anion clusters as can be seen from the stacking diagram (Figure S2, Supplementary Materials).

**Figure 2:**
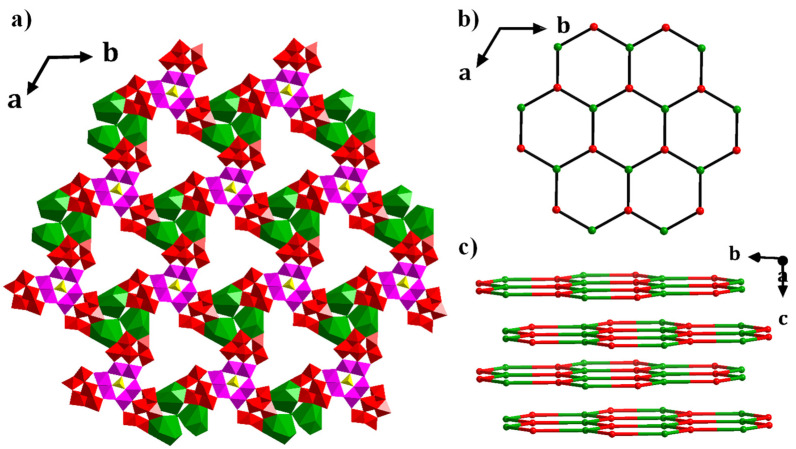
(**a**) Polyhedral view of **1** along the *c*-axis. Color code: WO_6_: red, CoO_6_: pink, PO_4_: yellow, Cl@{Cs_3_(H_2_O)_6_}: green; (**b**) the topological network of **1** along the *c*-axis. (HPO_4_)_2_@{Co_9_(PW_9_)_3_} 3-c node: red; Cl@{Cs_3_(H_2_O)_6_} 3-c node: green; (**c**) the 2D layers are arranged in –ABAB– manner along the *c*-axis.

From a topological point of view, **1** can be simplified as a three-connected 2D network (Figure 2a), in which (HPO_4_)_2_@{Co_9_(PW_9_)_3_} (red nodes) and [Cl@{Cs_3_(H_2_O)_6_}] (green nodes) can be simplified as three-connected (3-c) nodes (Figure 2b), respectively. Interestingly, the 2D layers are arranged in an –ABAB– manner along the *c*-axis (Figure 2c).

### 3.2. IR Spectrum and Optical Band Gap

FT-IR spectra were measured in the range of 400–4000 cm^−1^ with KBr pallets (Figure S3, Supplementary Materials). The strong peaks at around 3430 and 1630 cm^−1^ are dominated by the stretching and bending modes of the water molecules. The characteristic bands derived from the Keggin POM fragments in the 721–1030 cm^−1^ region. In detail, the peak at 1030 and 933 cm^−1^ for **1** is attributable to ν(P-Oa) and ν(W-Ot). The peak at 806, 721, and 692 cm^−1^ is attributable to ν(M-O-M) (M = W or Co), respectively. UV–Vis absorption and optical diffuse reflectance spectra of **1** were obtained in the wavelength range of 200–800 nm (Figure S4, Supplementary Materials). Absorption peaks of 260 nm and 535 nm correspond to the charge transfer between O→W and the *d-d* charge transfer between Co^2+^, respectively (illustration). The band-gap energy (*Eg*) is obtained by extrapolating the linear part of the rising curve to zero [32]. The *Eg* of 1 was estimated as 2.58 eV.

### 3.3. PXRD and Thermogravimetric Analysis

The pure phase was confirmed by powder X-ray diffraction (PXRD) patterns (Figure S5, Supplementary Materials). Thermogravimetric analysis was conducted under air flowing with the heating rate of 10 °C min^−1^ from 25 to 1000 °C (Figure S6, Supplementary Materials). The TG curve indicates that the weight of **1** shows one step loss of 10.25% (calcd 10.3%) from 25 to 600 °C, corresponding to the release of 40 lattice water molecules. six coordination water molecules, three hydroxy groups (1.5 H_2_O) and six protons (3 H_2_O) for **1**, respectively.

### 3.4. Photocatalytic H_2_ Evolution Performance

Herein, the three-component system was examined: [Ir(ppy)_2_(dtbbpy)][PF_6_] (ppy = 2-phenylpyridine; dtbbpy = 5,5′-di-tert-butyl-2,2′-bipyridine) as a photosensitizer, triethanolamine (TEOA) as sacrificial electron donor, and **1** as water reduction catalyst (WRC). The device used for the photocatalytic reaction is a multichannel photocatalytic reactor (Beijing Perfectlight Technology, Beijing, China), which uses white LED lamp beads (400–800 nm, electric power: 10 W), and is continuously illuminated for 10 h at a temperature of 25 °C. The reaction system was deaerated for 20 min under an Ar/CH_4_ (4:1) atmosphere. H_2_ was analyzed using a GC979011 gas chromatograph (Fuli Instruments, Taizhou, China) with a TCD and a 5 Å molecular sieve column (3 m × 3 mm) with Ar as the carrier gas.

**Figure 3:**
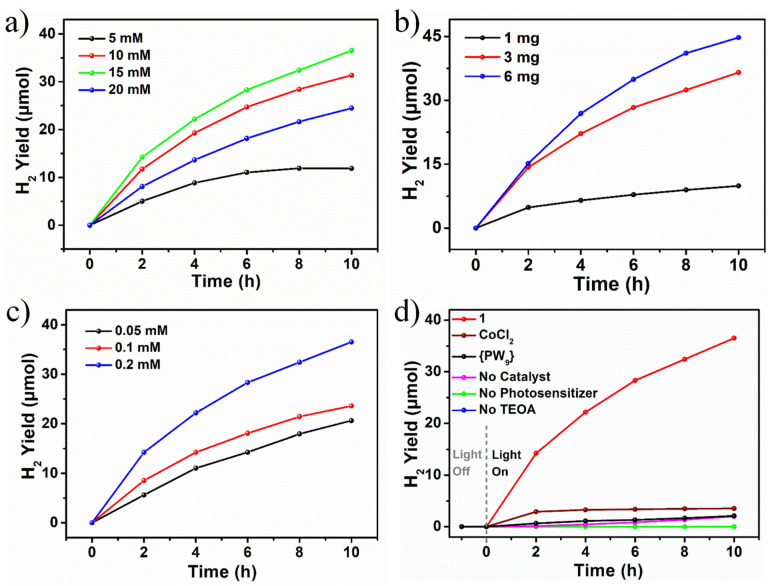
Photocatalytic H_2_ Evolution performance of **1**: (**a**) Time-dependent H_2_ yield of different TEOA concentrations (5–20 mM) of **1**. Reaction conditions: **1** (3 mg), white light (400–800 nm, 10 W), [Ir(ppy)_2_(dtbbpy)][PF_6_] (0.2 mM), 6 mL CH_3_CN/DMF (1:3) and H_2_O (2 M) degassed with Ar/CH_4_ (4:1); (**b**) Time-dependent H_2_ yield of different amounts of **1** (1~6 mg); (**c**) Time-dependent H_2_ yield of different concentrations of [Ir(ppy)_2_(dtbbpy)][PF_6_]; (**d**) Time-dependent H_2_ yield of light source and blank control under the condition of equimolar amount of 1, {PW_9_} and CoCl_2_.

As shown in Figure 3a, the H_2_ production increases with increasing radiation time. With the increasing concentrations of TEOA (5 mM, 10 mM, 15 mM, 20 mM) in the reaction system, the production of H_2_ showed a trend of first increasing (11.88 μmol, 31.36 μmol, 36.53 μmol, respectively) and then decreasing (24.48 μmol) after 10 h of illumination. One possible explanation is that the addition of TEOA increases the pH value of the system, reducing the concentration of H^+^ in the system, and thereby reducing the catalytic effect. The H_2_ production (9.9 μmol, 36.5 μmol and 40.7 μmol, respectively) increases with the increasing quantity of catalyst (1, 3, 6 mg), and the H_2_ evolution rate was 745.6 μmol h^−1^ g^−1^, 1217.6 μmol h^−1^ g^−1^ and 745.6 μmol h^−1^ g^−1^, respectively (Figure 3b). In terms of catalytic efficiency, the maximum H_2_ production rate of 3 mg can reach 1217.6 μmol h^−1^ g^−1^. Considering the catalytic system is heterogeneous catalysis, with the increase in the amount of catalyst, part of the light is scattered and the catalytic efficiency decreases. Hence, efficient catalysis may be achieved using the appropriate amount of catalyst. When the concentration of the photosensitizer (0.05 mM, 0.1 mM, 0.2 mM) was changed under the optimal conditions, the amount of the H_2_ production was 20.6 μmol, 23.58 μmol and 36.53 μmol, respectively, and the amount of hydrogen production increased with the increase in the concentration of the photosensitizer (Figure 3c). The PXRD and FT-IR (Figure S7, Supplementary Materials) tests before and after the photocatalytic reaction proved that the structure of **1** was basically unchanged and had good catalytic stability.

For heterogeneous catalytic systems, the stability and recyclability of catalysts are always important factors. In the cycling experiments (Figure S8, Supplementary Materials), the yield of H_2_ was detected by GC analysis, and the catalyst was isolated by centrifugation and washed with CH_3_CN after two hours of reaction at a catalyst dosage of 9 mg. The experiments were repeated for three successive cycles, the amount of H_2_ production was similar in the first two cycles but decreased the third time. Such a decrease in activity might be ascribed to the loss of the catalyst sample during the isolating operation. The PXRD pattern and FT-IR spectra (Figure S9, Supplementary Materials) of the catalyst before and after cycling experiments proved that **1** remains unchanged, which further demonstrated the recyclability and stability of complex **1**.

In conclusion, the best reaction conditions explored were: **1** (3 mg), TEOA (15 mM) and photosensitizer (0.2 mM), and the best H_2_ production efficiency can reach 1217.6 μmol h^−1^ g^−1^. This value is comparable to that of previously reported results for H_2_ evolution (Table S3, Supplementary Materials). Considering the factors of catalyst efficiency, 3 mg **1** was used for in-depth studies. As shown in Figure 3d, H_2_ was produced only when the photosensitizer [Ir(ppy)_2_(dtbbpy)]-[PF_6_] and TEOA electron sacrificial reagent were added with light irradiation, and a small amount of H_2_ was produced when the equimolar amount of {PW_9_} and CoCl_2_·6H_2_O was used as the control catalyst revealing the superiority of CoAPs in the visible-light-driven H_2_ evolution. According to the reported literature [22,33,34], a possible photocatalytic mechanism for this visible-light-driven H_2_ evolution process was proposed (Figure S10, Supplementary Materials). In visible-light-driven catalytic systems, the photosensitizer’s excited state can function as either an oxidant or reductant and thus can be quenched by an electron donor or an acceptor. A POMs-based catalyst and TEOA can oxidatively and reductively quench the excited state of [Ir(ppy)_2_(dtbbpy)]^+^ * in the visible-light-driven H_2_ evolution process catalyzed by **1**.

### 3.5. Magnetic Properties

**Figure 4:**
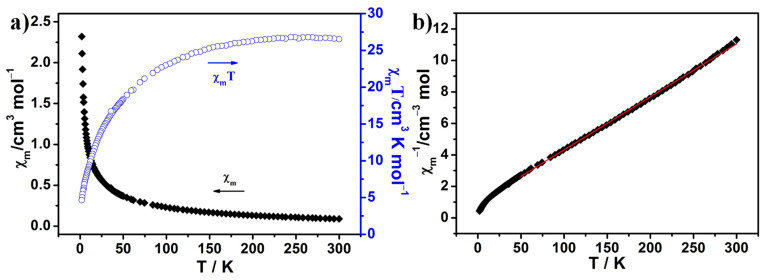
(**a**) Temperature dependence of *χ_m_* and *χ_m_T* values for **1** at 5000 Oe; (**b**) temperature dependence of *χ_m_*^−1^ values for **1** at 5000 Oe. The red line is fit to the Curie–Weiss law.

The variable-magnetic properties of **1** were measured in the temperature range of 2–300 K with a magnetic field of 5000 Oe. As shown in Figure 4a, the *χ_m_T* value is 26.53 cm^3^ mol^−1^ K at 300 K. The *χ_m_T* value decreases continuously with decreasing temperature. When the temperature drops below 50 K, the *χ_m_T* value decreases rapidly. The *χ_m_T* drops to 4.64 cm^3^ mol^−1^ K at 2 K. The above behaviors suggest antiferromagnetic interactions in **1**. The magnetic susceptibility data of **1** are in accordance with the Curie–Weiss law *χ* = *C*/(*T*−*θ*) (Figure 4b) in the temperature range of 50–300 K with a constant of *θ* = −28.25 K, which is consistent with the overall antiferromagnetic interaction in **1**.

## 4. Conclusions

In conclusion, a polyanion (HPO_4_)_2_@{Co_9_(PW_9_)_3_} cluster was made with the guidance of a lacunary directing strategy under hydrothermal conditions. The HPO_4_ of **1** originates from the central heteroatom of the tri-lacunary precursor [A-*α*-PW_9_O_34_]^9−^ and assembles with a cyclic (HPO_4_)_2_@{Co_9_} cluster to form CoAP **1**. Variable-magnetic properties indicate antiferromagnetic interactions in **1**. Moreover, visible-light-driven H_2_ evolution tests demonstrated that **1** is an eco-friendly, efficient and stable WRC with an H_2_ evolution rate of 1217.6 μmol h^−1^ g^−1^. This work further proves that the lacunary directing strategy is an effective guideline for the synthesis of TMAPs under hydrothermal conditions. The photocatalytic performance showed that CoAP is an efficient WRC. In future work, the application of POMs in heterogeneous catalytic reactions is in progress.

## Figures and Tables

**Table 1:** Crystal data and structure refinements for **1**.

	1
Formula	H_103_Na_8_ClCs_3_Co_9_O_158_P_5_W_27_
Mr	8898.75
Crystal system	Hexagonal
Space group	*P*6_3_/*m*
*a* [Å]	20.7840(8)
*b* [Å]	20.7840(8)
*c* [Å]	20.6385(9)
*α* [º]	90
*β* [º]	90
*γ* [º]	120
*V* [Å^3^]	7721.1
*Z*, *D*c [g cm^−3^]	2
*F*(000)	7316.0
GOF on *F*^2^	1.036
Final *R* indices [*I* > 2σ(*I*)]	*R*_1_ = 0.0762 *wR*_2_ = 0.2026
*R* indices [all data] ^a^	*R*_1_ = 0.01328 *wR*_2_ = 0.2464
CCDC number	2213769

^a^ R1 = Σ||F_0_| − |F_c_||/Σ|F_0_|. wR_2_ = [Σw(F^2^_0_ − F^2^c)^2^/Σw(F^2^_0_)^2^]^1/2^.

## Data Availability

Not applicable.

## Supplementary Materials

The following supporting information can be downloaded at: https://www.mdpi.com/article/10.3390/molecules28020664/s1, Table S1: Bond valence sum (BVS) calculations of all the W, P, Co, O, and Cl atoms in **1**; Figure S1: View of the asymmetric unit of **1**. Symmetry codes: A (x, y, 1.5-z), B (1-y, 1+x-y, z); Figure S2: (**a**) Polyhedral view of compound **1** along the c-axis; (**b**) Ball-and-stick model of the Cl@{Cs_3_(H_2_O)_6_} cluster; Figure S3: FT-IR spectra of **1**; Figure S4: UV–Vis spectra of **1**; Figure S5: Simulated and experimental powder X-ray diffraction patterns of **1**; Figure S6: TG curves of **1**; Figure S7: Stability test of **1** after catalysis: (**a**) PXRD patterns before and after photocatalysis; (**b**) FT-IR patterns before and after photocatalysis; Figure S8: Yield of H_2_ for **1** (9 mg) as a photocatalyst in three continuous runs; Figure S9: Stability test of **1** after three-time recycling: (**a**) The powder X-ray diffraction patterns after three-time recycling of catalyst **1**; (**b**) The FT-IR spectra after three-time recycling of catalyst **1**; Figure S10: Proposed mechanism for visible-light-driven H_2_ evolution tests by **1** with oxidative and reductive quenching mechanism; Table S2: Visible-light-driven H_2_ evolution catalyzed by different TMAP-based photocatalysts. References [14,15,35,36,37] are cited in the Supplementary Materials.

## Sample Availability

Samples of the compounds are available from the authors.
