# Supplementary material for: A {Co9}-Added Polyoxometalate for Efficient Visible-Light-Driven Hydrogen Evolution
Source: Molecules. 2023 Jan 9;28(2):664. doi: 10.3390/molecules28020664 (PMC9861855; doi:10.3390/molecules28020664)
Supplement: Supplementary file 1 [file molecules-28-00664-s001.zip › molecules-2063617-supplementary.pdf]

Supplementary Materials

A {Co_9}-Added Polyoxometalate for Efficient Visible-Light-Driven Hydrogen Evolution

Zhen-Wen Wang, and Guo-Yu Yang *

MOE Key Laboratory of Cluster Science, School of Chemistry and Chemical Engineering, Beijing Institute of Technology, Beijing 102488, China; wzw@bit.edu.cn (Z.-W.W)
* Correspondence: ygy@bit.edu.cn or ygy@fjirsm.ac.cn)

Table S1. Bond valence sum (BVS) calculations of all the W, P, Co, O, and Cl atoms in **1**.
Figure S1. View of the asymmetric unit of **1**. Symmetry codes: A (x, y, 1.5-z), B (1-y, 1+x-y, z).
Figure S2. (a) Polyhedral view of compound **1** along the c-axis; (b) Ball-and-stick model of the Cl@{$Cs_3(H_2O)_6$} cluster.
Figure S3: FT-IR sperctra of **1**.
Figure S4: UV-Vis sperctra of **1**.
Figure S5: Simulated and experimental powder X-ray diffraction patterns of **1**.
Figure S6: TG curves of **1**.
Figure S7: Stability test of **1** after catalysis: (**a**) PXRD patterns before and after photocatalysis; b) FT-IR patterns before and after photocatalysis.
Figure S8. Yield of H_2 for **1** (9 mg) as a photocatalyst in three continuous runs.
Figure S9. Stability test of **1** after three times recycle: (**a**) The powder X-ray diffraction patterns after three times recycle of catalyst **1**; (**b**) The FT-IR spectra after three times recycle of catalyst **1**.
Figure S10: Proposed mechanism for visible-light-driven H_2 evolution tests by **1** with oxidative and reductive quenching mechanism.
Table S2. Visible-light-driven H_2 evolution catalyzed by different TMAP-based photocatalysts.

Table S1. Bond valence sum (BVS) calculations of all the W, P, Co, O, and Cl atoms in **1**.

Atom	BVS	Atom	BVS
P1	4.827	P2	5.048
W1	6.026	W2	6.324
W3	6.151	W4	5.593
W5	5.843	Co1	2.094
Co2	1.961	O1	1.299
O2	2.232	O3	1.084
O4	1.835	O5	1.903
O6	1.955	O7	2.033
O8	1.749	O9	1.973
O10	1.765	O11	2.173
O12	1.808	O13	2.061
O14	1.890	O15	1.883
O16	1.778	O17	1.992
O18	1.825	O19	1.639
O20	1.762	O21	1.907
O22	1.917	O23	1.887
Cl1	0.888		

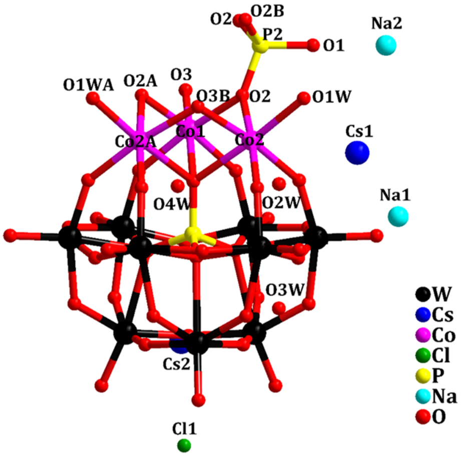

Figure S1. View of the asymmetric unit of **1**. Symmetry codes: A (x, y, 1.5-z), B (1-y, 1+x-y, z).

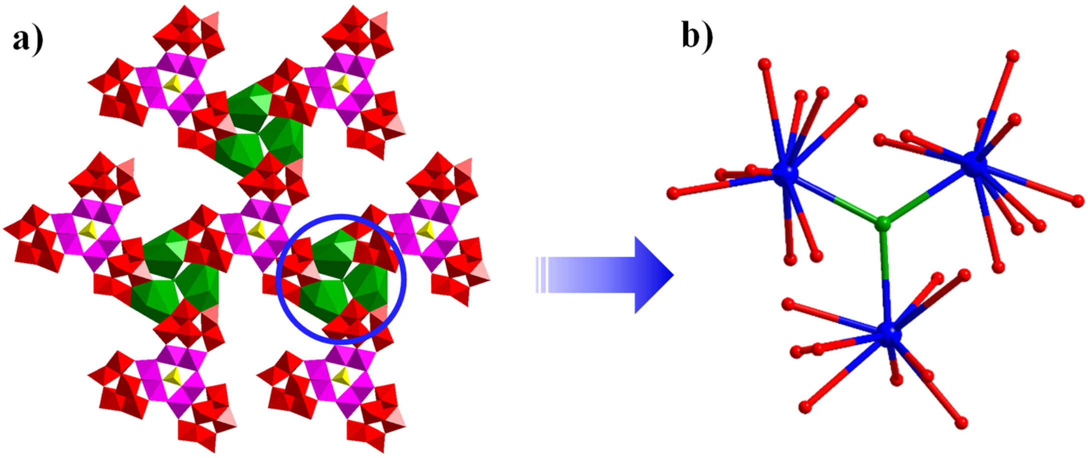

Figure S2. (**a**) Polyhedral view of **1** along the c-axis; (**b**) Ball-and-stick model of the Cl@{$Cs_3(H_2O)_6$} cluster.

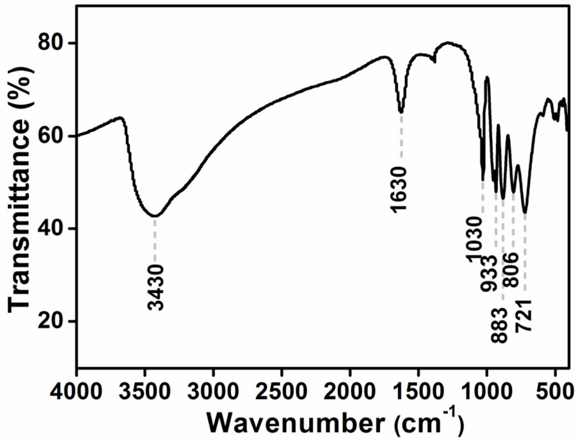

Figure S3. FT-IR sperctra of **1**.

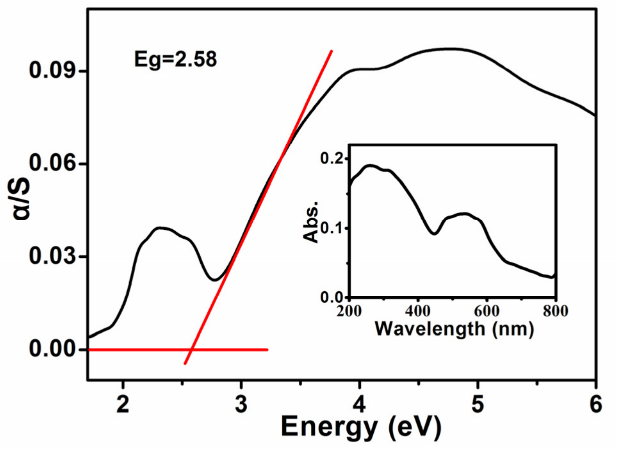

Figure S4. UV-Vis sperctra of **1**.

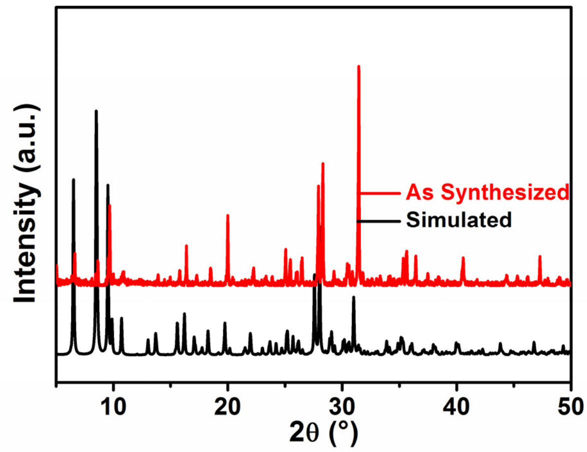

Figure S5. Simulated and experimental powder X-ray diffraction patterns of **1**.

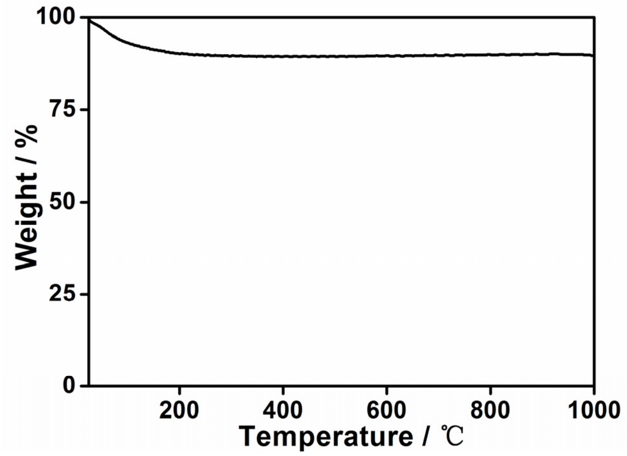

Figure S6. TG curves of **1**.

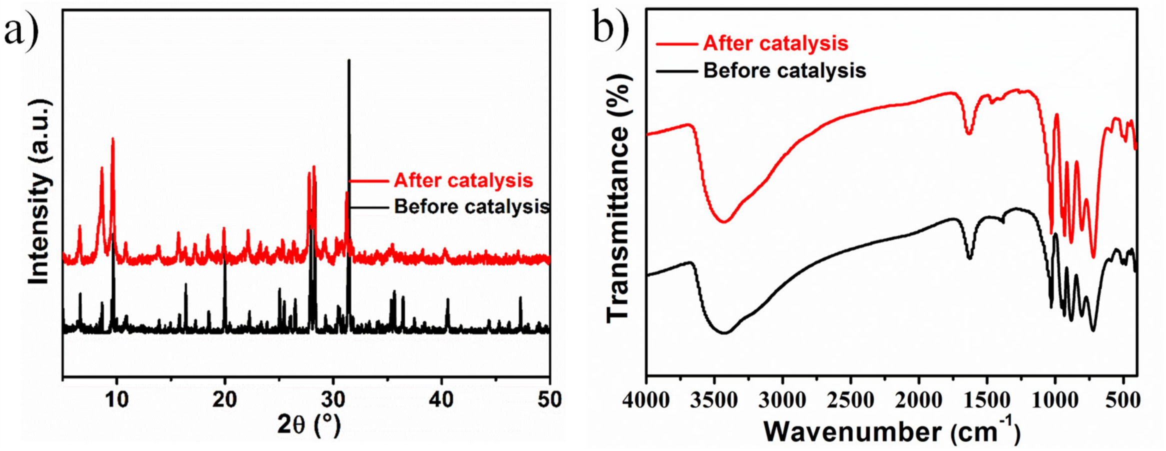

Figure S7. Stability test of **1** after catalysis: (**a**) PXRD patterns before and after photocatalysis; b) FT-IR patterns before and after photocatalysis.

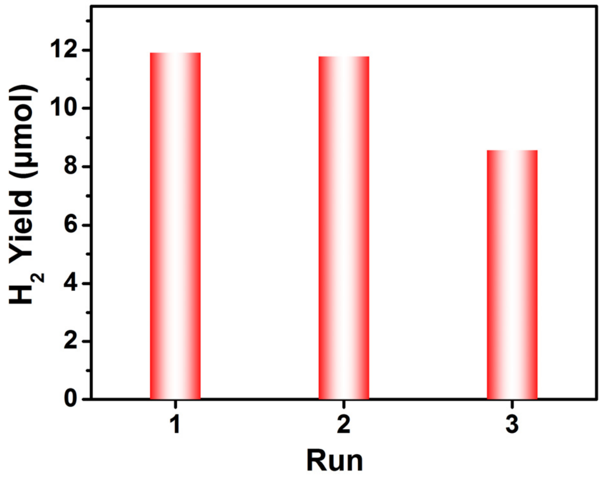

Figure S8. Yield of H_2 for **1** (9 mg) as a photocatalyst in three continuous runs.

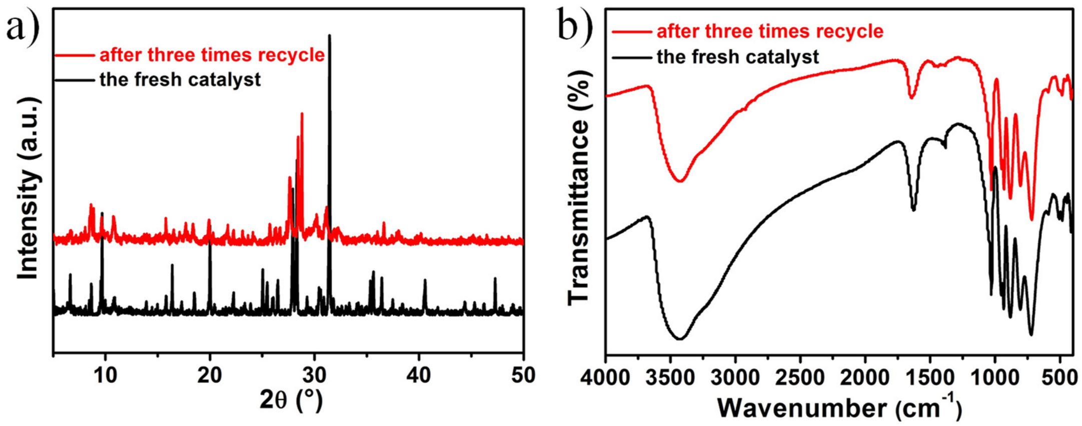

Figure S9. Stability test of **1** after three times recycle: (**a**) The powder X-ray diffraction patterns after three times recycle of catalyst **1**; (**b**) The FT-IR spectra after three times recycle of catalyst **1**.

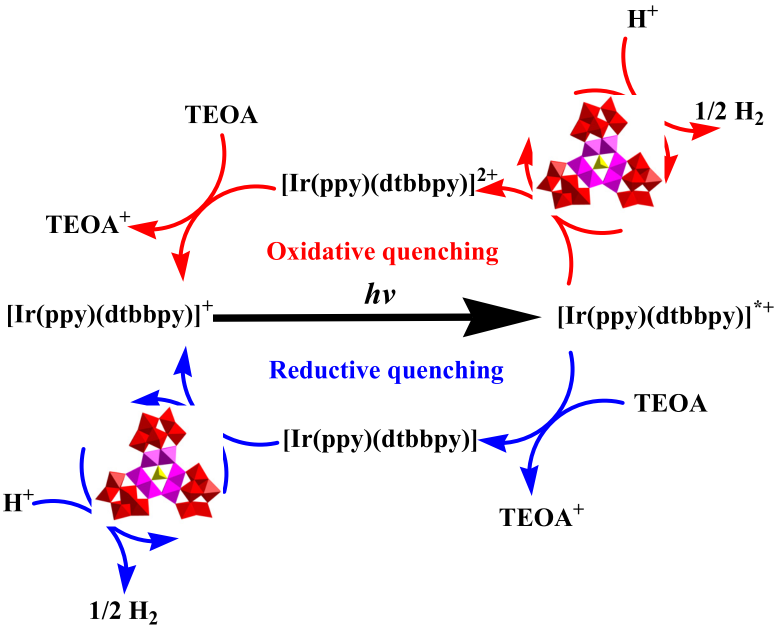

Figure S10. Proposed mechanism for visible-light-driven H_2 evolution tests by **1** with oxidative and reductive quenching mechanism.

Table S2. Visible-light-driven H_2 evolution catalyzed by different TMAP-based photocatalysts.

Catalyst	Light source	Photosensitizer	Cocatast	Sacrificial agent	Activity (μmol h^{-1} g^{-1})	Ref.
**1**	white LED light (10 W)	$[Ir(ppy)_2(dtbbpy)][PF_6]$	None	TEOA	1217.6	This work
$SiW_{11}Cu$	500 W Xe lamp with a 420 nm cut-off filter	None	Pt	zinc powders	150	1
$SiW_{11}Ni$					98	
$SiW_{11}Co$					65	
$SiW_{11}Zn$					48	
$TiW_{11}Fe$	Simulated solar light (350-760 nm)	None	Pt	PVA	500	2
$TiW_{11}Co$					484	
$TiW_{11}Zn$					294	
$[Cu_8(H_2O)_2(en)_4(B\text{-}\alpha\text{-}H_2SiW_9O_{34})_2]$	LED light (450 nm)	$[Ir(ppy)_2(dtbbpy)][PF_6]$	None	TEOA	833.3	3
$[(btc)Ni_6(\mu_3\text{-}OH)_3(H_2O)_5(B\text{-}\alpha\text{-}PW_9O_{34})]^{3-}$	white LED light (5 W)	$[Ir(ppy)_2(dtbbpy)][PF_6]$	None	TEOA	1058.2	4
$[Ni(trz)_3]_2@[Ni_{30}(H_2O)_{16}]$ POM-**1**	300 W Xe lamp with a 420 nm cut-off filter	$[Ir(ppy)_2(dtbbpy)][PF_6]$	None	TEOA/ BNAH	1216.7	5
$[Ni(trz)_3]_2@[Ni_{30}(H_2O)_{16}]$ POM-**2**					813.3	
$[Ni(trz)_3]_2@[Ni_{30}(H_2O)_{16}]$ POM-**4**					1036.7	

References

1. Wang, Z.; Lu, Y.; Li, Y.; Wang, S.; Wang, E. Visible-Light Photocatalytic H_2 Evolution over a Series of Transition Metal Substituted Keggin-Structure Heteropoly Blues. *Chin. Sci. Bull.* **2012**, *57*, 2265–2268.
2. Shang, X.; Liu, R.; Zhang, G.; Zhang, S.; Cao, H.; Gu, Z. Artificial Photosynthesis for Solar Hydrogen Generation over Transition-Metal Substituted Keggin-Type Titanium Tungstate. *New J. Chem.* **2014**, *38*, 1315–1320.
3. Sun, J.J.; Wang, W.D.; Li, X.Y.; Yang, B.F.; Yang, G.Y. $\{Cu_8\}$ Cluster-Sandwiched Polyoxotungstates and Their Polymers: Syntheses, Structures, and Properties. *Inorg. Chem.* **2021**, *60*, 10459–10467.
4. Wang, S.S.; Kong, X.Y.; Wu, W.; Wu, X.Y.; Cai, S.; Lu, C.Z. Synergic Coordination of Multicomponents for the Formation of a $\{Ni_{30}\}$ Cluster Substituted Polyoxometalate and Its In-Situ Assembly. *Inorg. Chem. Front.* **2022**, *9*, 4350–4358.
5. Wang, Z.W.; Zhao, Q.; Chen, C.A.; Sun, J.J.; Lv, H.; Yang, G.Y. Chiral $\{Ni_6PW_9\}$ Cluster–Organic Framework: Synthesis, Structure, and Properties. *Inorg. Chem.* **2022**, *61*, 7477–7483.
